# In Vitro Anti-Photoaging and Skin Protective Effects of *Licania macrocarpa* Cuatrec Methanol Extract

**DOI:** 10.3390/plants11101383

**Published:** 2022-05-23

**Authors:** Kon Kuk Shin, Sang Hee Park, Hye Yeon Lim, Laura Rojas Lorza, Nurinanda Prisky Qomaladewia, Long You, Nur Aziz, Soo Ah Kim, Jong Sub Lee, Eui Su Choung, Jin Kyung Noh, Dong-Keun Yie, Deok Jeong, Jongsung Lee, Jae Youl Cho

**Affiliations:** 1 Department of Integrative Biotechnology, Biomedical Institute for Convergence at SKKU (BICS), Sungkyunkwan University, Suwon 16419, Korea; shuka337@naver.com (K.K.S.); laurisrl@gmail.com (L.R.L.); priskyqomaladewi@gmail.com (N.P.Q.); youlonghc@gmail.com (L.Y.); nuraziz@skku.edu (N.A.); jd279601@gmail.com (D.J.); 2 Department of Biocosmetics, Sungkyunkwan University, Suwon 16419, Korea; 84701@naver.com (S.H.P.); gosl177@naver.com (H.Y.L.); 3 DanjoungBio Co., Ltd., Wonju 26303, Korea; sa.kim@danjoungbio.com (S.A.K.); js.lee@danjoungbio.com (J.S.L.); esavella@hanmail.net (E.S.C.); 4 Instituto de BioEconomia, El Batan, Quito 170135, Ecuador; njk1201@hotmail.com; 5 International Biological Material Research Center, Korea Research Institute of Bioscience and Biotechnology, Daejeon 34141, Korea; ydian78@kribb.re.kr; 6 Convergence Research Center for Energy and Environmental Sciences, Sungkyunkwan University, Suwon 16419, Korea

**Keywords:** UVB exposure, wrinkle formation, melanogenesis, HaCaT cells, B16 melanoma cells

## Abstract

The *Licania* genus has been used in the treatment of dysentery, diabetes, inflammation, and diarrhea in South America. Of these plants, the strong anti-inflammatory activity of *Licania macrocarpa* Cuatrec (Chrysobalanaceae) has been reported previously. However, the beneficial activities of this plant on skin health have remained unclear. This study explores the protective activity of a methanol extract (50–100 μg/mL) in the aerial parts of *L. macrocarpa* Cuatrec (Lm-ME) and its mechanism, in terms of its moisturizing/hydration factors, skin wrinkles, UV radiation-induced cell damage, and radical generation (using RT/real-time PCR, carbazole assays, flowcytometry, DPPH/ABTS, and immunoblotting analysis). The anti-pigmentation role of Lm-ME was also tested by measuring levels of melanin, melanogenesis-related genes, and pigmentation-regulatory proteins. Lm-ME decreased UVB-irradiated death in HaCaT cells by suppressing apoptosis and inhibited matrix metalloproteinases 1/2 (MMP1/2) expression by enhancing the activity of extracellular signal-regulated kinase (ERK), c-Jun N-terminal kinase (JNK), and p38. It was confirmed that Lm-ME displayed strong antioxidative activity. Lm-ME upregulated the expression of hyaluronan synthases-2/3 (HAS-2/3) and transglutaminase-1 (TGM-1), as well as secreted levels of hyaluronic acid (HA) via p38 and JNK activation. This extract also significantly inhibited the production of hyaluronidase (Hyal)-1, -2, and -4. Lm-ME reduced the melanin expression of microphthalmia-associated transcription factor (MITF), tyrosinase, and tyrosinase-related protein-1/2 (TYRP-1/2) in α-melanocyte-stimulating hormone (α-MSH)-treated B16F10 cells via the reduction of cAMP response element-binding protein (CREB) and p38 activation. These results suggest that Lm-ME plays a role in skin protection through antioxidative, moisturizing, cytoprotective, and skin-lightening properties, and may become a new and promising cosmetic product beneficial for the skin.

## 1. Introduction

The skin acts as a barrier that separates the body from the external world and protects it from infection and damage [1]. In addition, the skin also maintains the balance of our body electrolyte, temperature, and sensation [2]. Keratinocytes are abundant in the skin epidermis and use desmosomes and tight junctions to be connected to each other, which form the protective barrier [3]. Although the skin can act as a barrier to protect us, it can still be damaged by ultraviolet B radiation (UVB), with waves ranging from 290 to 32.0 nm [3,4,5]. UVB results in DNA damage and induces reactive oxygen species (ROS), which causes inflammation and damage to our skin, otherwise known as photoaging [6]. Moreover, UVB irradiation can not only lead to cell cycle arrest as well as apoptosis, but also causes degradation of matrix metalloproteinase (MMP)-related genes in the extracellular matrix. If our skin undergoes too much UVB irradiation, dysfunctional apoptosis can occur, which can cause some skin diseases, such as psoriasis, and even lead to skin cancer. Apart from that, MMP-related genes, such as MMP-1, priorly decomposes fibrillary collagens that keep the tensile strength of fetal membranes. Meanwhile, MMP-2 can cause the degradation of collagen types I and IV [7]. Therefore, protective effects are required against photo-related skin aging and diseases associated with UVB radiation.

UVB radiation causes dehydration of the skin tissue, which breaks down the skin barrier and causes skin aging [8]. Therefore, hydration is important for maintaining the normal functioning of the skin. In particular, the water content of the stratum corneum is important for proper maturation and skin desquamation [9]. The stratum corneum has protective features to mitigate water loss, including corneocytes, natural moisturizing factor (NMF), and lipid bilayer matrix. At the final stages of keratinocyte differentiation, there can cause disruption between plasma membrane and organelles (including the nucleus). Next, transglutaminase (TGM)-1 was stimulated by calcium influx and caused a crosstalk with loricrin and involucrin. Because of this, the cell envelope was formed with hard and insoluble characteristics. Meanwhile, it covered the keratin fibers in the resulting corneocyte. Hence, TGM is crucial for interacting with other proteins. A lack of TGM-1 contributes to the disorder of the skin, which causes lamella ichthyosis. In addition, insufficient water in the subcutaneous can lead to the dysfunction of corneocyte adhesion and an accumulation of corneocytes [10]. Hyaluronic acid (HA) shows a pivotal role in water retention. Membrane-bound enzymes, including hyaluronan synthase (HAS)-1, -2, and -3, increase the biosynthesis of HA [11].

The skin also contains melanocytes that produce melanin in epidermal units. Melanosomes are produced by melanocytes through a complex pathway, called melanogenesis, which is managed by microphthalmia-associated transcription factor (MITF) and its transcribed genes, such as tyrosinase, tyrosinase-related protein-1 (TRP-1), and TRP-2 [12]. Melanin is a dark pigment synthesized in melanocytes by oxidizing L-tyrosine to protect the skin from external stimuli such as UV rays. The main role of melanin is to protect the skin tissue from UV. However, when produced excessively, melanin causes skin pigmentation [13]. Pigmentation disorders can be divided into two parts: hyperpigmentation and hypopigmentation. Hyperpigmentation can induce melasma, while hypopigmentation can lead to oculocutaneous albinism (OCA) [14]. In the Asian world, white skin is considered a reflection of youth and beauty; therefore, many cosmetics seek to whiten the skin [15]. Therefore, melanin secretion and skin whitening are important in both disease and cosmetics fields.

The genus *Licania* includes more than 200 species that are mostly distributed in South America, from Panama to Peru [16]. The *Licania* genus has been used in the treatment of dysentery, diabetes, inflammation, and diarrhea in South America [17]. Besides these applications, the other characteristics of genus *Licania* related to skin health have not been investigated. Because of this, the properties of methanol extract of *Licania macrocarpa* Cuatrec (Chrysobalanaceae) (Lm-ME) on skin hydration, apoptosis, and melanogenesis with human keratinocytes and murine melanoma cells were studied.

## 2. Results

### 2.1. Antioxidatve Activity of Lm-ME and Its Phytochemical Fingerprinting Profiles

**Figure 1:**
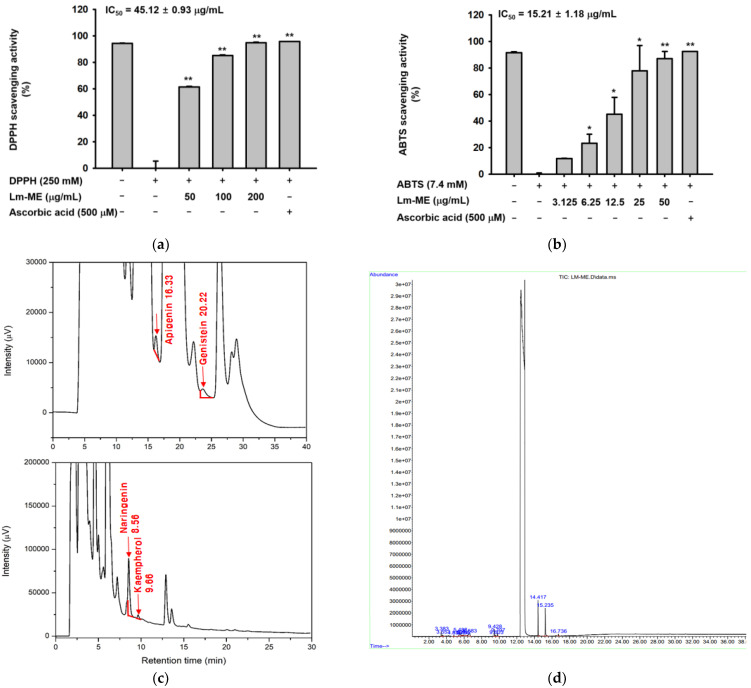
The antioxidative activity of Lm-ME and its phytochemical fingerprinting profiles. (**a**,**b**) DPPH and ABTS assays were performed to confirm the ROS scavenging activity of Lm-ME. Ascorbic acid (500 μM) was used as a positive control. (**c**) The phytochemical fingerprinting profiles of Lm-ME using HPLC analysis. (**d**) Qualitative analysis of Lm-ME using GC/MS analysis. *: *p* < 0.05 and **: *p* < 0.01 compared to the control group (DPPH or ABTS alone).

To determine whether Lm-ME can protect human keratinocytes from reactive oxygen species induced by UVB irradiation, DPPH and ABTS assays were performed to evaluate the effect of Lm-ME on antioxidant activity. As a result, IC_50_ values of LM-ME were measured to be 45.12 (DPPH) and 15.21 (ABTS) μg/mL. These results show that LM-ME has potent antioxidant activity. Ascorbic acid (500 μM) was used as a positive control (Figure 1a,b). The phytochemical fingerprinting of Lm-ME (100 μg/mL), using HPLC analysis with standard flavonoids of apigenin, genistein, naringenin, kaempferol, hesperidin, and rutin, (Figure 1c), as reported previously [16,18], was also quantified. Indeed, apigenin (59.1 ppm), genistein (116.8 ppm), naringenin (1346.6 ppm), and kaempferol (20.8 ppm) were identified using HPLC analysis, in addition to previously identified quercetin [17]. The phytochemical composition of LM-ME was further analyzed by gas chromatography–mass spectrometry (GC-MS) (Figure 1d). Table 1 shows all 14 compounds contained in LM-ME.

### 2.2. Lm-ME Showed the Role of UVB Protection on Human Keratinocytes

**Figure 2:**
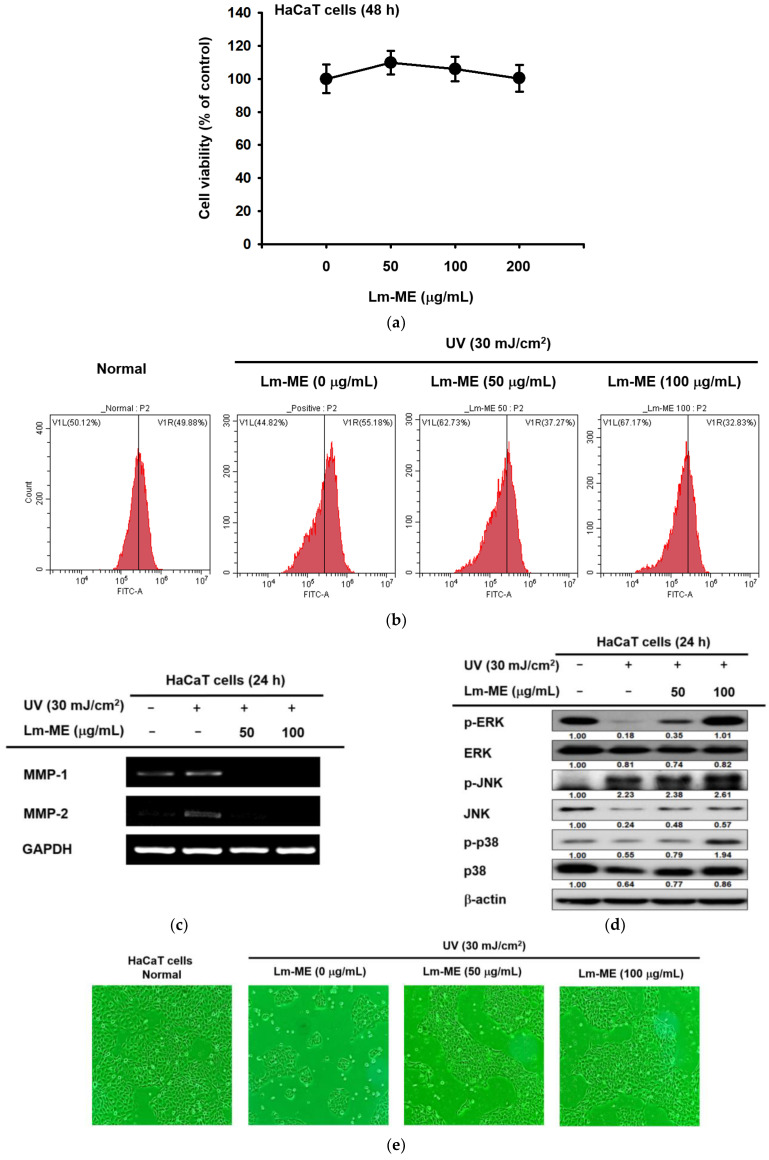

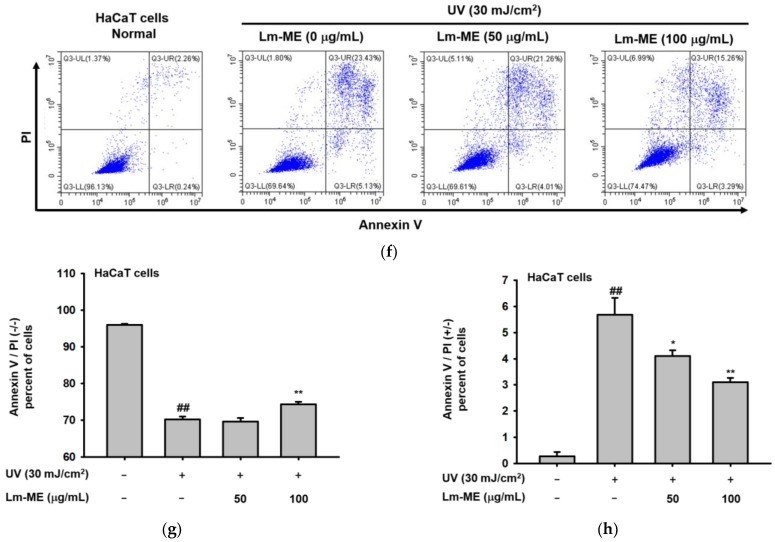
The protective effects of Lm-ME under UVB irradiation. (**a**) HaCaT cells were treated with Lm-ME at 50 and 100 μg/mL. After 24 h, cell viability was measured using the MTT assay. (**b**) HaCaT cells were stained with DCFDA, which were evaluated with flow cytometry to check ROS production. (**c**) The mRNA expression of MMP-1 and -2 was analyzed using RT-PCR. GAPDH was used as an internal control. (**d**) After UVB induction (30 mJ/cm^2^), cells were treated with dose-dependently Lm-ME (0–100 μg/mL) for 24 h. Phosphorylated and total ERK, JNK, and p38 were tested with Western blot. (**e**) HaCaT cells were pretreated with Lm-ME and continuously irradiated with UVB. After 48 h, photos of cells were taken using a digital camera. (**f**–**h**) HaCaT was stained with annexin V/PI and detected using flow cytometry. The bottom values of c and d indicate the relative intensity measured using ImageJ. ##: *p* < 0.01 compared to the normal group and *: *p* < 0.05 and **: *p* < 0.01 compared to the control group.

To check whether Lm-ME can protect UVB-induced cellular responses, several cellular responses were studied using HaCaT cells. First, the cytotoxicity-inducing activity of this extract was examined. As Figure 2a shows, Lm-ME did not reduce the viability of HaCaT cells. Next, flow cytometry was employed to analyze the antioxidant effects of Lm-ME in UVB-irradiated HaCaT cells. ROS was stained with dichlorodihydrofluorescein diacetate (DCFDA) under UV conditions. In fact, it was found that treatment with Lm-ME decreased the level of ROS induced by UV irradiation (50–100 μg/mL) (Figure 2b). In addition, after UV irradiation (30 mJ/cm^2^), the production of MMP-1 and -2 clearly increased in HaCaT cells (Figure 2c). However, Lm-ME dramatically downregulated the transcriptional levels of MMP-1 and -2 induced by UVB treatment. Glyceraldehyde 3-phosphate dehydrogenase (GAPDH) was used as an internal control (Figure 2c). Lm-ME inhibited ROS activity in a concentration-dependent manner (Figure 2b), suggesting that Lm-ME ameliorates apoptosis under UV conditions through its antioxidant effects. It is known that antioxidants can suppress apoptosis by reducing ROS production [19]. While the levels of phospho-ERK, JNK and p38 were down-regulated under UV induction conditions, Lm-ME treatment recovered the phosphorylation levels of ERK, JNK, and p38 (Figure 2d). Then, the recovery activity of Lm-ME in HaCaT cells, which were exposed to UVB, was also examined. Interestingly, Lm-ME was found to restore cell death caused by UVB (Figure 2e). Additionally, flow cytometry was also applied to analyze the effects of Lm-ME under UV-induced apoptosis, stained by annexin V/PI. Lm-ME increased the percentage of annexin V/PI (−/−) in double-negative cells, whereas annexin V/PI (+/−) in single-positive cells was suppressed by Lm-ME (Figure 2f–h). Therefore, Lm-ME treatment relieved apoptosis, but enhanced cell viability.

### 2.3. The Effects of Lm-ME on Hydration/Moisturization of HaCaT Cells

**Figure 3:**
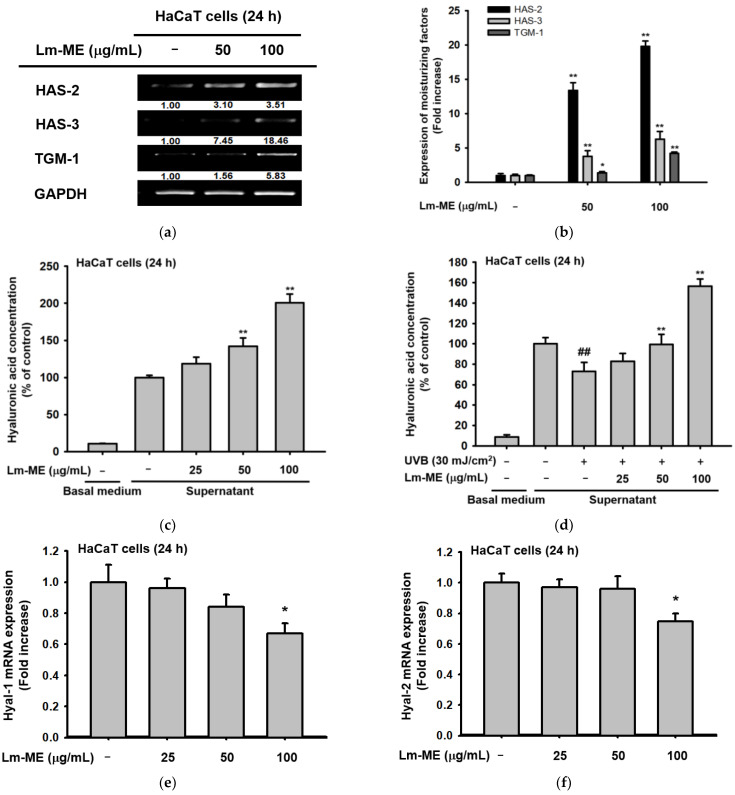

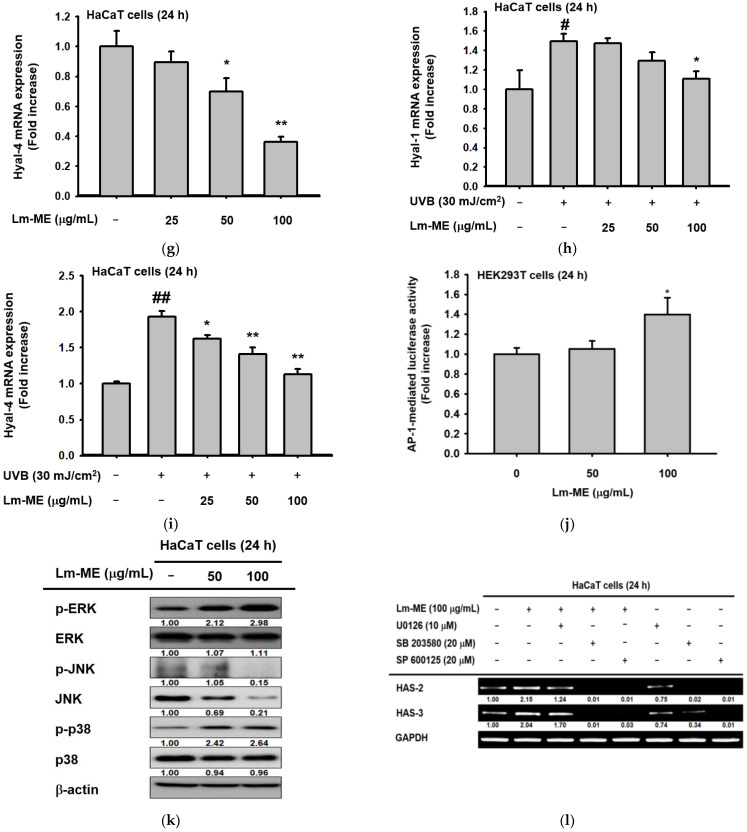
The hydration effects of Lm-ME in HaCaT cells. (**a**,**b**) After treatment with Lm-ME for 24 h, the hydration-related genes (HAS-2, -3, and TGM-1) were analyzed in HaCaT cells by semi-quantitative and real-time PCR. GAPDH was used as an internal control. (**c**,**d**) Hyaluronic acid levels were determined from culture supernatants of normal Lm-ME-treated or UVB-irradiated HaCaT cells using the carbazole assay. (**e**–**i**) The mRNA expressions of Hyal-1, -2, and -4 were determined from normal Lm-ME-treated or UVB-irradiated HaCaT cells using real-time PCR. (**j**) The AP-1 luciferase construct and β-gal (as control) were transfected for 24 h. The HEK293T cells were then treated with Lm-ME at different concentrations (0–100 μg/mL). Luciferase activity was measured using a luminometer. (**k**) The HaCaT cells were treated with Lm-ME (0–100 μg/mL) for 24 h. The levels of phosphorylated, as well as the total ERK, JNK, and p38 gene products, were tested using immunoblotting. (**l**) The HaCaT cells were then treated with Lm-ME (0–100 μg/mL) and MAPK inhibitors, such as U 0126, SB 203580, and SP 600125. After this treatment, the mRNA expression levels of HAS-2 and HAS-3 were analyzed using semi-quantitative PCR. The relative intensity of bottom values of a, k, and l are measured using ImageJ. #: *p* < 0.05 and ##: *p* < 0.01 compared to the normal group, and *: *p* < 0.05 and **: *p* < 0.01 compared to the control group.

Hyaluronic acid (HA) is a dominant moisturizing ingredient in skin, which largely distributed in both dermis and epidermis. Hyaluronic acid synthase (HAS) is a kind of gene that can accumulate intermediate-sized HA in keratinocytes [11], whereas TGM-1 is located in the spinous and granular layers of the skin. In epidermal keratinocytes, TGM-1 facilitates crosslinking of the cell envelope [20]. HaCaT cells were treated with Lm-ME. After 24 h, the mRNA levels of HAS-1, -2, and TGM-1 were enhanced (Figure 3a). A similar induction pattern of HAS-2, HAS-3, and TGM-1 was confirmed using real-time PCR (Figure 3b). The HA content assay, with a dye carbazole, was used to elucidate whether HA production can also be increased by Lm-ME in normal and UVB-irradiated HaCaT cells (Figure 3c,d). Lm-ME dose-dependently elevated the level of HA in normal HaCaT cells and completely recovered HA levels decreased by UVB irradiation. Interestingly, Lm-ME was revealed to suppress the expression levels of HA hydrolysis enzymes. Thus, Lm-ME downregulated the expressions of Hyal-1, -2, and -4 from normal HaCaT cells (Figure 3e–g). Moreover, this extract also suppressed the expression of Hyal-1 and Hyal-4, but not Hyal-2 and Hyal-3, which were upregulated by UVB irradiation (Figure 3h,i). HAS-1 and HAS-2 are associated with mitogen-activated protein kinase (MAPK) signaling, which promotes HAS-1 and HAS-2 gene expression (especially via the p38 pathway) [21]. The AP-1 luciferase assay increased the Lm-ME (100 μg/mL) up to 1.399-fold compared to the normal group (Figure 3j). Western blot analysis of MAPK was also conducted under Lm-ME-treated conditions. Lm-ME increased the phospho-ERK and p38 (Figure 3k), implying that this extract can regulate these enzymes. To determine whether these enzymes can contribute to the induction of HAS-2 and HAS-3 gene expression by Lm-ME, inhibitors of p38 (SB203580) and ERK (U0126) were employed by assessing the expression levels of HAS-2 and HAS-3. SB203580, but not U0125, strongly suppressed the upregulation of HAS-2 and HAS -3 gene transcription, implying that Lm-ME-upregulated by p38 can play a critical role in HAS gene expression (Figure 3l).

### 2.4. Lm-ME Suppressed Pigmentation Induced by α-MSH in B16F10 Cells

**Figure 4:**
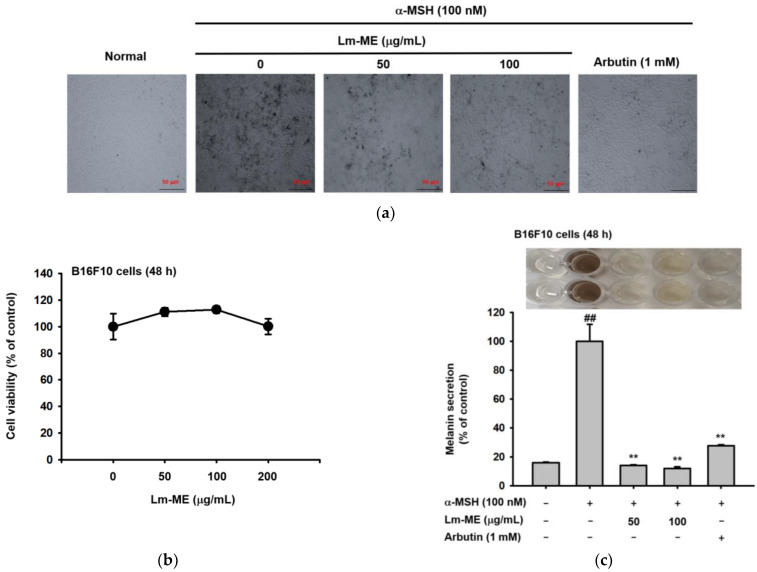

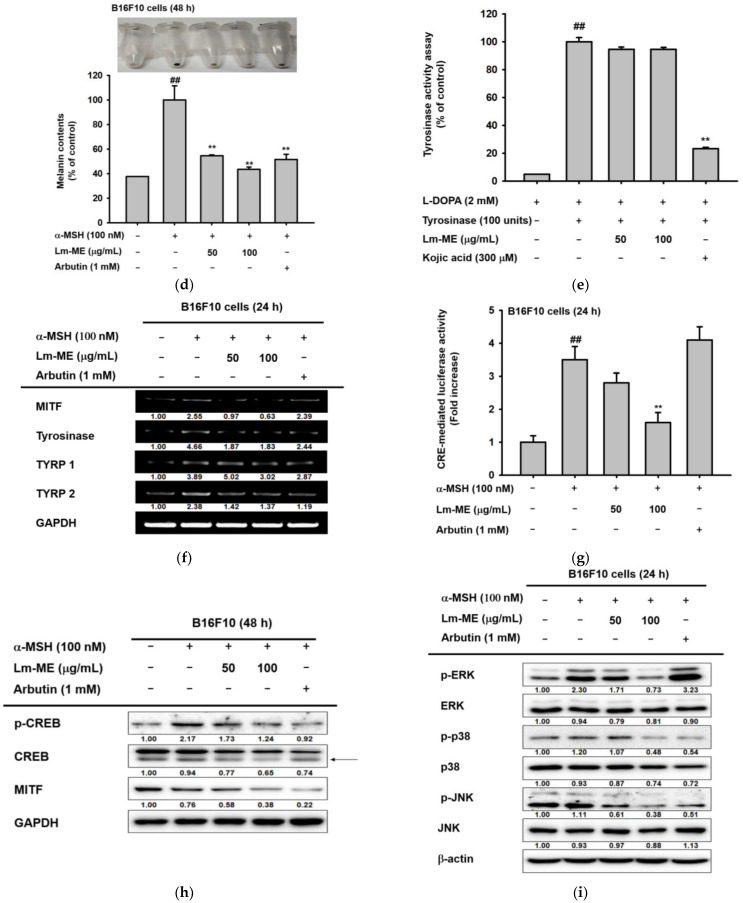
The anti-pigmentation effects of Lm-ME. (**a**) B16F10 cells were co-treated with α-MSH Lm-ME (0–100 μg/mL) or arbutin (1 mM) for 48 h. Photos were taken using a Nikon Eclipse Ti inverted microscope (DS-Qi1Mc, Nikon Shinagawa, Tokyo, Japan). (**b**) The B16F10 cells were treated with Lm-ME (0–100 μg/mL) for 48 h. The cell viability was examined with an MTT assay. (**c**) B16F10 cells were induced using α-MSH for 48 h. Lm-ME (0–100 μg/mL) and arbutin (1 mM), as the control for 48 h. Melanin secretion was tested at 475 nm. (**d**) To measure the melanin content, the B16F10 cell pellets were lysed using a lysis buffer. The absorbance of B16F10 cell lysates was measured at 405 nm using spectrophotometry. (**e**) Tyrosinase activity was induced via mushroom tyrosinase (100 U/mL). Furthermore, 2 mM of L-DOPA and Lm-ME (0–100 μg/mL) or kojic acid (300 μM) was co-treated for 15 min. Next, mushroom tyrosinase was added. The absorbance of each sample was measured at 475 nm. (**f**) B16F10 cells were co-treated with α-MSH and Lm-ME or arbutin for 24 h. The mRNA levels of melanogenesis-related genes were checked by RT-PCR. GAPDH was used as an internal control. (**g**) The CREB-luc and β-gal were transfected with PEI for 24 h. The B16F10 cells were then treated with Lm-ME in a dose-dependent manner. CREB luciferase activity was checked using a luminometer. (**h**) The B16F10 cells were co-treated with α-MSH and Lm-ME or arbutin for 48 h. The total and phosphorylated forms of MITF and CREB were measured using Western blot. (**i**) The B16F10 cells were treated with α-MSH and Lm-ME or arbutin for 24 h. Next, the total and phospho-ERK, JNK, and p38 were tested using Western blot. The bottom values of (**f**,**h**,**i**) indicate the relative intensity measured using ImageJ (Wayne Rasband, NIH, Bethesda, MD, USA). ##: *p* < 0.01 compared to the normal group, and **: *p* < 0.01 compared to the control group.

B16F10 cells were stimulated using α-MSH for 48 h to produce melanin. Compared to only treatment of α-MSH, cells treated with Lm-ME (100 μg/mL) and arbutin (1 mM) showed less melanin in the cells (Figure 4a). Meanwhile, Lm-ME (50–200 μg/mL) did not exhibit cytotoxicity in cells from the MTT assay (Figure 4b). Similar data also show that α-MSH (100 nM) treatment created B16F10 cells darkened the media color, whereas the melanin secretion and content were both decreased by Lm-ME. (Figure 4c,d). Next, the tyrosinase activity assay was performed to determine whether the inhibition of Lm-ME is due to the direct suppression of enzymatic activity. However, tyrosinase activity was not affected by Lm-ME, while kojic acid (300 μM) did affect tyrosinase activity (Figure 4e), implying indirect inhibition. Continuously, the transcriptional levels of *MITF*, *tyrosinase*, *TYRP1*, and *TYRP2* were measured. All the mRNA levels of the aforementioned genes were decreased by Lm-ME 100 μg/mL (Figure 4f). Since MITF can induce the transcription of TYRP 1 and TYRP 2 in melanogenesis to produce eumelanin and pheomelanin [13], the molecular inhibitory mechanism of Lm-EE’s anti-melanogenic responses were further investigated. Figure 4g,h show that Lm-ME was able to block CREB-mediated luciferase activity and phosphorylation of this transcription factor. Because CREB activity can be controlled by MAPK [22], the phospho-protein levels of ERK, JNK, and p38 were measured (Figure 4i). Lm-ME (100 μg/mL) blocked phosphorylation levels of all MAPKs, indicating their roles in Lm-ME-mediated anti-melanogenesis.

## 3. Discussion

The health of the skin is affected by many factors, including UV irradiation, oxidative stress, and dehydration [23]. For instance, UV-activated p53 can lead to cell apoptosis and DNA damage. Therefore, UV irradiation can be carcinogenic [10]. Oxidative stress induced by UV irradiation can interfere with genome maintenance and induce apoptosis [23]. The skin becomes dehydrated due to excessive skin lipid removal in several skin diseases, including psoriasis, atopic dermatitis, and xerotic eczema [24]. Therefore, the integrity of the skin barrier is important for human health. This can be achieved by protecting the skin from UV irradiation and ROS, and by maintaining its hydration. Firstly, the anti-apoptosis effect of Lm-EE under the UVB irradiation was evaluated. In a previous paper, quercetin and kaempferol were found in the methanol extract of *L. macrocarpa* Cuatrec [16]. Plenty of papers have discussed the antioxidant properties of quercetin and kaempferol [25,26]. In addition, other flavonoids, such as apigenin (59.1 ppm), genistein (116.8 ppm), and naringenin (1346.6 ppm), were also identified (Figure 1c). Synthetically, the methanol extract of *L. macrocarpa* Cuatrec, with various kinds of flavonoid compounds, was revealed to have antioxidant effects that protect against apoptosis caused by UV radiation (Figure 2f–h).

UV irradiation can upregulate MMP genes in HaCaT cells, including MMP-1 and -2 [27]. Our compound inhibited the transcription level of MMP to block ECM (Figure 2c). Moreover, the production of MMP-1 from keratinocytes induces fibroblasts to secrete inflammatory cytokines, such as IL-1β and IL-6 [28]. One strategy to prevent fibroblast photoaging, which is caused by the relationship between keratinocytes and fibroblasts, is to inhibit MMP in keratinocytes. On the contrary, Lm-ME can also increase the transcription level of skin moisturizing factors, such as HAS-2, HAS-3, and TGM-1 (Figure 3a,b). These genes regulate keratinocytes and hydrate the skin. Active forms of mitogen-activated protein kinases (MAPKs), such as the phosphorylation of ERK and p38, were induced by Lm-ME (Figure 3k). According to analysis with MAPK inhibitors (Figure 3l), p38 was revealed to modulate the expression of hydration-related genes. Another group found that p38 has a critical role in regulating hyaluronan synthases 1 and 2 [29,30]. Therefore, our data and other group reports strongly suggest that p38 phosphorylation influences hydration in HaCaT cells during Lm-ME exposure. In addition, ERK inhibition can lead to apoptosis in HaCaT cells [31,32]. However, since Lm-ME increased ERK activation, ERK could contribute to the anti-apoptosis effects of Lm-ME.

UV irradiation induces DNA damage, sequentially activating p53. Activated p53 promotes the transcription of α-MSH in keratinocytes [3,33]. In turn, α-MSH triggers melanocortin 1 receptor (MC1R) activation in melanocytes, leading to the stimulation of the cAMP-dependent signaling pathway, which can activate CREB and induce MITF transcription [34]. As a result, MITF stimulates melanogenesis-related genes, such as tyrosinase genes (TYR), namely TYRP1 and TYRP2 [13]. In Figure 4g,h, the luciferase assay showed the apparent inhibition of CREB activation, and immunoblotting analysis exhibited the respective suppression of p-CREB under Lm-ME-treated conditions. These data imply that the CREB pathway is critically inhibited in the Lm-ME-mediated suppression of melanogenesis, as reported for other chemicals showing anti-melanogenic activities, such as *Patrinia villosa* (Thunb.) Juss ethanol extract, rottlerin, and beauvericin [35,36]. However, the MAPK signal is complex because the role of MAPKs is still controversial. For example, ERK and p38 activation can decrease MITF expression and melanogenesis [3,37], while suppression of these enzymes increases MITF levels [38,39]. From the data, it is evident that α-MSH can significantly enhance the phosphorylation of ERK and p38, while Lm-ME reduced their phosphorylation (Figure 4h). It is suggested that ERK inhibition seems to further contribute to the suppression of the melanogenic response by Lm-ME. However, more research is necessary to further understand the role of MAPK in melanogenic signal transduction, with particular emphasis on the co-inhibition of ERK and p38 by overexpression of ERK and p38, as well as the knockdown of each. Because melanogenesis is a time-sensitive process, these future studies may improve our understanding of MAPK and melanogenesis. This could also lead us to make a conclusion on the role of ERK in Lm-ME-mediated anti-melanogenic activity.

## 4. Materials and Methods

### 4.1. Materials

The leaves and stems of *L. macrocarpa* Cuatrec, obtained from the International Biological Material Research Center (Daejeon, Korea), were extracted by methanol (Lm-ME; code no: FBM160-098) [16]. HaCaT cells were purchased form Cell Lines Service (CLS) GmbH (Eppelheim, Germany). B16F10 cells were obtained from the American Type Culture Collection (Rockville, MD, USA). The cell culture media (highly modified DMEM and high-glucose DMEM) and FBS were purchased from Hyclone (Grand Island, NY, USA). The other cell culture media (F-10 and Opti-MEM) were purchased from Gibco (Grand Island, NY, USA). 5-hydroxy-2-hydroxymethyl-4H-pyranone (kojic acid), L-3,4-dihydroxyphenylalanine (L-DOPA), monophenol monooxygenase (mushroom tyrosinase), 1-diphenyl-2 picryl-hydrazyl (DPPH), 2,2′-azino-bis (3-ethylbenzothiazoline-6-sulphonic acid) diammonium salt (ABTS), ascorbic acid (AA), polyethylenimine (PEI), α-melanocyte-stimulating hormoneα (α-MSH), dimethylsulfoxide (DMSO), 3-(4,5-dimethylthiazol-2′,7′-dichlorofluorescein diacetate (DCFDA), and 3-(4,5-dimethylthiazole-2-yl)-2,5-diphenyltetrazolium bromide (MTT) were purchased from Sigma Chemical Co. (St. Louis, MO, USA). The kit of the luciferase reporter gene assay containing CREB-binding promoters was obtained from Promega (Madison, WI, USA). The TRIzol reagent was purchased from Thermo Fisher Scientific (Waltham, MA, USA). The PCR premix was purchased from Bio-D Inc. (Seoul, Korea). The antibodies of ERK, JNK, p38, CREB, MITF, and β-actin were purchased from Cell Signaling Technology (Beverly, MA, USA). The annexin V/PI kit was purchased from BD Biosciences (San Jose, CA, USA).

### 4.2. Cell Culture

HaCaT cells were cultured in DMEM with 10% heat-inactivated FBS and 1% antibiotics. B16F10 cells were cultured in F10 media with 10% FBS and 1% antibiotics. All cells were incubated in 5% CO_2_ incubator at 37 °C.

### 4.3. Compound Treatment

The powder of Lm-ME (25.93 g) was dissolved in DMSO to make a stock at the concentration of 50 mg/mL. When used for in vitro experiments, Lm-ME was diluted to 50–100 μg/mL with culture media. The same amount of DMSO was included in the control group.

### 4.4. High-Performance Liquid Chromatography and Gas Chromatography–Mass Spectrometry Analyses

We took high-performance liquid chromatography (HPLC) to identify the ingredients of Lm-ME. Several standards were used, such as genistein, quercetin, and kaempferol [22,40]. The analyzing conditions are shown in Table 2. Naringenin (67604-48-2, TCI), kaempferol (520-18-3, Sigma), genistein (446-72-0, TCI), apigenin (320-36-5, Sigma), hesperidin (520-26-3, Sigma), and rutin (153-18-4, Sigma) were used to create standard curves. GC-MS analysis of Lm-ME was carried out using the Cooperative Center for Research Facilities of Sungkyunkwan University (Suwon, Korea), as previously reported [41].

### 4.5. MTT Assay

To evaluate cell viability, the MTT assay was carried out using stopping solution (10% sodium dodecyl sulfate containing 1 M HCl) [42,43]. Cells were seeded in 96-well plates around 20 h. Then, cells were added to 50–100 μg/mL of Lm-ME for 24 h. Afterwards, 10 μL of MTT was added into 96-well plates. After incubating for 3 h, 100 μL of MTT stopping solution was injected. Sixteen to twenty hours later, the plates were measured at 570 nm using a microplate reader, as previously reported [43].

### 4.6. UVB Irradiation

HaCaT cells were seeded into 6-well plates with the density of 4 × 10^5^ cells/well. The medium was then removed and covered with 1 mL of PBS. Then, the cells were exposed to a UVB lamp (Bio-Link BLX-312; Vilber Lourmat, Collégien, France) at 312 nm, as previously reported [44]. Later, the Lm-ME was re-treated to each well, respectively.

### 4.7. UVB-Induced Apoptosis Analysis

To detect apoptosis levels, flow cytometry staining with annexin V/PI was employed. HaCaT cells were exposed to UVB, as described above. Firstly, the cells were washed twice with cold PBS. Then, the cells were diluted by binding buffer until the concentration reached 1 × 10^6^ cells/mL. Next, 100 μL cells were stained using propidium iodide (PI) and fluorescein isothiocyanate–annexin V at room temperature for 15 min. Later, the cells were washed twice with 500 μL of staining buffer and Beckman CytoFLEX Flow Cytometer (Beckman Coulter, Brea, CA, USA) was used to measure fluorescence.

### 4.8. Cellular ROS Analysis

2′,7′–dichlorofluorescin diacetate (DCFDA) was used to detect ROS [45]. HaCaT cells were irradiated with UVB and resuspended with 300 μL of PBS. Furthermore, 10 μM DCFDA was added in the tube to incubate in 37 °C for 30 min. The fluorescence was detected at 485/535 nm using a flow cytometer.

### 4.9. DPPH Assay

The scavenging effect of Lm-ME was assessed using the DPPH assay [46,47]. Lm-ME (50–200 μg/mL) and ascorbic acid (500 μM) were mixed with 250 mM of DPPH in a dose-dependent manner for 25 min at room temperature. Then, we measured the absorbance at 517 nm. Ascorbic acid (500 μM) was used as a positive control. The inhibition was calculated as follows: DPPH scavenging effect (%) = (Ab0 − Ab1)/A0 × 100, in which Ab0 is the absorbance of DPPH, and Ab1 is the absorbance of samples.

### 4.10. ABTS Assay

The ABTS assay was also conducted as reported [47,48]. Moreover, 7.4 mM of ABTS and 2.4 mM of potassium persulfate were mixed before the experiments at a ratio of 1:1. Lm-ME (3.125–50 μg/mL) and ascorbic acid (500 μM) were added together with ABTS solution at a ratio of 1:19. Then, the mixture was detected at 730 nm using a microplate reader. Ascorbic acid (500 μM) was used as a positive control The percentage of scavenging was calculated the same using the DPPH assay.

### 4.11. Semi-Quantitative Reverse Transcriptase-Polymerase Chain Reaction (RT-PCR) and Quantitative Real-Time PCR

HaCaT cells were irradiated within or without UVB and treated with Lm-ME dose-dependently till 24 h. Similarly, B16F10 cells were stimulated with α-MSH for 24 h and then co-incubated with Lm-ME for another 24 h. Semi-quantitative RT-PCR was performed, as previously reported [49,50]. Relative band intensities were measured using the imageJ software (Wayne Rasband, NIH, Bethesda, MD, USA). These treatment protocols were also applied in real-time PCR. The primers of RT-PCR and real-time PCR are shown in Table 3.

### 4.12. Western Blot Analysis

B16F10 cells were gathered with cold PBS and lysed for 30 min on ice. Then, the lysate mixture was centrifuged at 12,000 rpm for 15 min at 4 °C. Protein concentrations were tested using the Bradford assay, and Western blotting was conducted, as reported [51]. Band intensities were measured using ImageJ software.

### 4.13. Carbazole Assay for Hyaluronan Quantification

HaCaT cells (1.5 × 10^5^ cells/mL) were spread in 12-well plates for 24 h. After that, they were treated with Lm-ME and UVB, or only Lm-ME, and incubated for 24 h. Then, the cultured supernatant was transferred to a new tube for 50 μL each. Next, 200 μL of 25 mM sodium tetraborate dissolved in sulfuric acid was added and reacted at 95 °C for 5 min. Then, 50 μL of 0.125% carbazole in ethanol was added into each tube for 10 min, and the absorbance at 530 nm was checked using a microplate reader (BioTek, Winooski, VT, USA).

### 4.14. Melanogenesis and Melanin Secretion

B16F10 cells were treated with α-MSH (100 nM) for 48 h within or without Lm-ME or arbutin. To analyze melanin secretion, the medium was incubated for 48 h and detected at 475 nm [52]. To evaluate the melanin content, the cells were harvested in e-tubes and washed with cold PBS twice. After that, lysis buffer was used with 20 μL for each tube. After centrifuging at 12,000 rpm for 5 min, the pellet of each tube was collected and dissolved in 10% DMSO of 1 M NaOH solution at 50 °C for 10 min. The absorbance was checked at 405 nm using a microplate reader.

### 4.15. Tyrosinase Assay

The tyrosinase assay was tested, as previously reported [53]. To assess the tyrosinase enzyme activity, we mixed 50 μL of 2 mM L-DOPA and Lm-ME in 50 mM potassium phosphate buffer (K_3_PO_4_, pH 6.8) for 15 min. Next, 50 μL of mushroom tyrosinase (100 U/mL) was mixed into the solution at room temperature. For the negative control, 50 μL of 2 mM L-DOPA was mixed with 50 μL of 50 mM potassium phosphate buffer. The absorbance of each sample was measured at 475 nm.

### 4.16. Luciferase Reporter Gene Assay

B16F10 cells were seeded in 24-well plates at the density of 0.8 × 10^5^ cells/well. After incubating for 18 h, the plasmid CREB-Luc gene and the β-galactosidase gene were co-transfected in the cells with polyethyleneimine (PEI) [49]. After transfecting for 24 h, the treatment was the same as melanogenesis, as previously reported [49,54]. Then, after incubating for 24 h, B16F10 cells were lysed with the lysis buffer. Next, all the steps were followed using the kit of the luciferase assay, purchased from Promega (Madison, MI, USA). Finally, the absorbance was checked at 475 nm using a microplate reader.

### 4.17. Statistical Analyses

All of the data were calculated as mean ± standard deviation (SD) of three independent experiments. For the statistical comparisons, the ANOVA/Scheffe’s post-hoc test or the Kruskal–Wallis/Mann–Whitney tests were applied. *p*-values < 0.05 were considered statistically significant.

## 5. Conclusions

**Figure 5:**
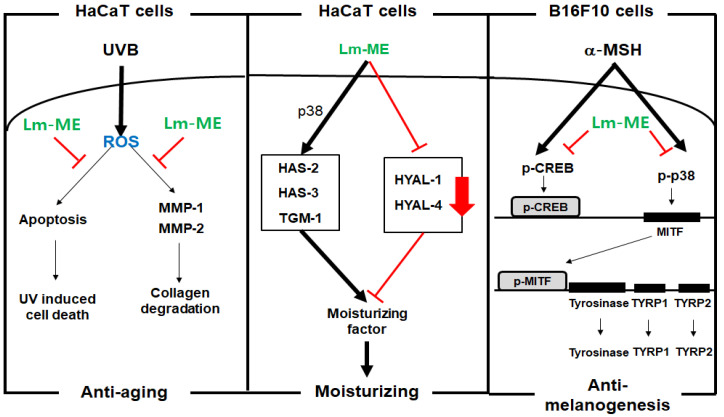
Schematic summary of the skin protective actions of Lm-ME with UV protection, moisturizing, anti-melanogenesis properties.

Lm-ME (50–100 μg/mL) was found to protect skin cells by ameliorating UV irradiation, enhancing moisturizing conditions, and controlling hyperpigmentation, as summarized in Figure 5. Thus, this extract suppressed apoptosis-induced cell death, MMP expression, and ROS generation in UVB-induced HaCaT cells. The strong antioxidative activity of this plant was also determined by DPPH and ABTS. Furthermore, Lm-ME could induce the expression of HAS-2 and -3 and TGM-1 via activation of p38. Treatment with Lm-ME reduced melanin production and mRNA expression of MITF, tyrosinase, and TYRP1/2 in α-MSH-treated B16F10 cells via the reduction of CREB and p38 activation. Taken together, our data strongly indicate that Lm-ME could be further developed for protective skin remedies.

## Figures and Tables

**Table 1:** Phytochemical analysis of Lm-ME.

Peak No.	RT	Name of the Compound	Corrected Area	% of Total
1	3.383	Piperidine	6,769,119	2.77%
2	3.553	Carbonic acid gas	6,631,886	2.71%
3	4.819	Ethanol	2,818,376	1.15%
4	5.376	Dimethyl sulfide	625,946	0.26%
5	5.488	2-Thiapropane	25,021,273	10.22%
6	5.756	Methylthiomethane	2,364,524	0.97%
7	5.912	Methyl sulfide	3,966,607	1.62%
8	6.583	Dimethyl monosulfide	27,990,880	11.44%
9	9.428	2,3-Dithiabutane	14,268,284	5.83%
10	9.603	Chloromethylmethyl sulfide	5,181,894	2.12%
11	9.797	Methanesulphinic acid methyl ester	7,917,119	3.23%
12	14.417	Dimethyl sulfone	71,638,885	29.27%
13	15.235	Methyl-d3-hydrazine sulfate	62,574,639	26.67%
14	16.736	S-Methyl methanethiosulphonate	4,303,891	1.76%

**Table 2:** Instrument and working conditions for HPLC.

Instrument	Condition A			Condition B		
Column	CAPCELL PAK C_18_ MG, 4.6 mm I.D. × 250 mm					
Detector	UV-Vis Detector					
Wavelength	254 nm			350 nm		
Compounds for analysis	Naringenin, kaempferol, and genistein			apigenin, hesperidin, and rutin		
Analyzed period	30 min			40 min		
Solvent	Solvent A	2% acetic acid in water		Solvent A	0.1% formic acid in MeOH:water = 10:90	
	Solvent B	0.5% acetic acid in water: ACN = 50:50		Solvent B	0.1% formic acid in MeOH:water = 90:10	
Flow rate	1 mL/min			0.4 mL/min		
Volume	10 μL			10 μL		
Gradient	Time (min)	Composition (%)		Time (min)	Composition (%)	
		A	B		A	B
	0	28	72	0	40	60
	20	0	100	20	40	60
	30	0	100	25	70	30
	-	-	-	40	70	30

**Table 3:** Sequences of primers used for PCR.

PCR Type	Gene Name	Sequence (5′-3′)	
RT-PCR (human)	MMP-1	F	TCTGACGTTGATCCCAGAGAGCAG
		R	CAGGGTGACACCAGTGACTGCAC
	MMP-2	F	AAAACGGACAAAGAGTTGGCA
		R	CTGGGGCAGTCCAAAGAACT
	HAS-2	F	CCACCCAGTACAGCGTCAAC
		R	CATGGTGCTTCTGTCGCTCT
	HAS-3	F	TTCTTTATGTGACTCATCTGTCTCACCG
		R	ATTGTTGGCTACCAGTTTATCCAAACG
	TGM-1	F	AGGGAAGATCCAAGAGCCCA
		R	ACTCTGGATCCCCTACGCTT
	HYAL-1	F	TGT GGA CGT GGA TGT CAG TG
		R	GTA GTA GGG GTA GGT GCC CA
	HYAL-2	F	ATG TGC AGA ACT GGG AGA GC
		R	GGA AGC AAG TGT CTC GTC CA
	HYAL-3	F	TCT GGG CAT CAT AGC CAA CC
		R	AGA GGC CGA GTT GGT TCT TG
	HYAL-4	F	TCC TGT GAT TGG AAG CCC AC
		R	TAA TGG GGA CCC CCT GTG AT
	GAPDH	F	GGTCACCAGGGCTGCTTTTA
		R	CACCGAGGAACTACCTGAT
Real-time PCR (human)	HYAL-1	F	TGTGGACGTGGATGTCAGTG
		R	GTAGTAGGGGTAGGTGCCCA
	HYAL-2	F	ATGTGCAGAACTGGGAGAGC
		R	GGAAGCAAGTGTCTCGTCCA
	HYAL-3	F	TCTGGGCATCATAGCCAACC
		R	AGAGGCCGAGTTGGTTCTTG
	HYAL-4	F	TCCTGTGATTGGAAGCCCAC
		R	TAATGGGGACCCCCTGTGAT
	HAS-2	F	TGACAGGCATCTCACGAACC
		R	TGGCGGGAAGTAAACTCGAC
	HAS-3	F	TATACCGCGCGCTCCAA
		R	GCCACTCCCGGAAGTAAGACT
	TGM-1	F	GAAATGCGGCAGATGACGAC
		R	AACTCCCCAGCGTCTGATTG
	GAPDH	F	GAC AGT CAG CCG CAT CTT CT
		R	GCG CCC AAT ACG ACC AAA TC
RT-PCR (mouse)	MITF	F	TCTGACGTTGATCCCAGAGAGCAG
		R	CAGGGTGACACCAGTGACTGCAC
	Tyrosinase	F	AAAACGGACAAAGAGTTGGCA
		R	CTGGGGCAGTCCAAAGAACT
	TYRP-1	F	ATGGAACGGGAGGACAAACC
		R	TCCTGACCTGGCCATTGAAC
	TYRP-2	F	CAGTTTCCCCGAGTCTGCAT
		R	GTCTAAGGCGCCCAAGAACT
	GAPDH	F	CACTCACGGCAAATTCAACGGCAC
		R	GACTCCACGACATACTCAGCAC

F: Forward; R: Reverse.

## Data Availability

The data are contained within the article.

## Abbreviations

Lm-ME	*Licania macrocarpa* Cuatrec methanol extract
CREB	cAMP response element (CRE)
DCDFA	2′,7′-dichlorodihydrofluorescein diacetate
ERK	extracellular signal-regulated kinase
JNK	c-Jun N-terminal kinase
MAPKs	mitogen-activated protein kinases
MMP	matrix metalloproteinases
MITF	microphthalmia-associated transcription factor
α-MSH	α-melanocyte-stimulating hormone
MTT	3-(4,5-dimethylthiazol-2-yl)-2,5-diphenyltetrazolium bromide
ROS	reactive oxygen species
RT-PCR	reverse transcription-polymerase chain reaction
TYRP-1	tyrosinase-related protein 1
TYRP-2	tyrosinase-related protein 2
UV	ultraviolet
HPLC	high-performance liquid chromatography
HA	hyaluronic acid
